# Model-based cardiovascular monitoring of large pore hemofiltration during endotoxic shock in pigs

**DOI:** 10.1186/cc9536

**Published:** 2011-03-11

**Authors:** JA Revie, DJ Stevenson, JG Chase, CE Hann, GM Shaw, A Le Compte, BC Lambermont, A Ghuysen, P Kolh, T Desaive

**Affiliations:** 1 University of Canterbury, Christchurch, New Zealand; 2 Christchurch Hospital, Christchurch, New Zealand; 3 University of Liege, Belgium

## Introduction

The aim of this research is to test the ability of a model-based method to track disease-dependent hemodynamic changes in sepsis. Thus, subject-specific models of the cardiovascular system (CVS) are identified using measurements from a porcine model of septic shock with hemofiltration [1].

## Methods

Hemodynamic measurements were recorded every 30 minutes in four (porcine model) trials of 4 hours. Animals received a 0.5 mg/kg endotoxin infusion over the first 30 minutes and underwent zero-balance continuous venovenous filtration with 0.7 m^2 ^large pore substrate (80 kDa cut-off) from 60 minutes onwards [1]. Subject-specific CVS models were fitted to 34 sets of data from the four trials. Each dataset represents a minimal set of measurements available in an ICU. Identified physiological model parameters and model outputs were compared with experimentally derived indices and measurements for validation.

## Results

The model predicted the left and right ventricular end-diastolic volumes and maximum left and right ventricular pressures to mean absolute errors of 7.1% and 6.7%. Changes in the modelled right ventricular end systolic elastance and pulmonary vascular resistance compared well (*R *= 0.68 and 0.73) with the same metrics derived experimentally (via caval occlusion manoeuvre and four-element Windkessel model) from an earlier study on right ventricular-vascular coupling [1]. Clinically, the systemic vascular resistance (SVR) model parameter decreased initially in all four pigs and stabilised to a level 26% (on average) below baseline during hemofiltration. Hyperdynamic states were observed in two pigs, where increases in left ventricle contractility were unable to counteract the loss in SVR, resulting in decreased mean arterial pressure (MAP) and increased cardiac output (CO) in the model, consistent with the experimental measurements. In contrast, for the other two pigs, increases in SVR after hemofiltration helped maintain MAP, with CO remaining relatively constant over the duration of these trials.

## Conclusions

Subject-specific CVS models are capable of accurately capturing acute disease-dependent hemodynamic changes due to endotoxic shock in pigs using a minimal set of measurements that are available in a typical ICU setting.
